# Cell-Type Specific Metabolic Response of Cancer Cells to Curcumin

**DOI:** 10.3390/ijms21051661

**Published:** 2020-02-28

**Authors:** Anamarija Mojzeš, Marko Tomljanović, Lidija Milković, Renata Novak Kujundžić, Ana Čipak Gašparović, Koraljka Gall Trošelj

**Affiliations:** 1 Laboratory for Epigenomics, Ruđer Bošković Institute, Division of Molecular Medicine, 10 000 Zagreb, Croatia; Anamarija.Mojzes@irb.hr (A.M.); Marko.Tomljanovic@irb.hr (M.T.); Renata.Novak.Kujundzic@irb.hr (R.N.K.); 2 Laboratory for Oxidative Stress (LabOS), Ruđer Bošković Institute, Division of Molecular Medicine, 10 000 Zagreb, Croatia; Lidija.Milkovic@irb.hr (L.M.); Ana.Cipak.Gasparovic@irb.hr (A.Č.G.)

**Keywords:** PKM1, PKM2, SHMT2, PHGDH, STAT3, serine, intracellular localization, ethanol

## Abstract

In order to support uncontrolled proliferation, cancer cells need to adapt to increased energetic and biosynthetic requirements. One such adjustment is aerobic glycolysis or the Warburg effect. It is characterized by increased glucose uptake and lactate production. Curcumin, a natural compound, has been shown to interact with multiple molecules and signaling pathways in cancer cells, including those relevant for cell metabolism. The effect of curcumin and its solvent, ethanol, was explored on four different cancer cell lines, in which the Warburg effect varied. Vital cellular parameters (proliferation, viability) were measured along with the glucose consumption and lactate production. The transcripts of pyruvate kinase 1 and 2 (PKM1, PKM2), serine hydroxymethyltransferase 2 (SHMT2) and phosphoglycerate dehydrogenase (PHGDH) were quantified with RT-qPCR. The amount and intracellular localization of PKM1, PKM2 and signal transducer and activator of transcription **3** (STAT3) proteins were analyzed by Western blot. The response to ethanol and curcumin seemed to be cell-type specific, with respect to all parameters analyzed. High sensitivity to curcumin was present in the cell lines originating from head and neck squamous cell carcinomas: FaDu, Detroit 562 and, especially, Cal27. Very low sensitivity was observed in the colon adenocarcinoma-originating HT-29 cell line, which retained, after exposure to curcumin, a higher levels of lactate production despite decreased glucose consumption. The effects of ethanol were significant.

## 1. Introduction

The prominent characteristic of malignant cells is the ability to adjust signaling and metabolic pathways to maintain well-known cancer hallmarks [1]. These pathways are interconnected in various ways and can be explored from many different points of view. The ability of cancer cells to sustain proliferative signaling needs to be studied in as many parameters as possible, including specific genetic background and metabolic profile. Uncontrolled proliferation is dependent on cellular metabolism rewiring to meet increased energetic and biosynthetic requirements, and orchestrate multiple signaling pathways central to cancer cell survival. The Warburg effect or aerobic glycolysis is considered as a crucial adaptive, metabolic response to the increased biosynthetic demand of cancer cells. It is characterized by an increased uptake of glucose and hyperproduction of lactate, even when sufficient oxygen is available. Highly proliferative cancer cells have an increased utilization of serine for protein synthesis, the synthesis of other amino acids, nucleotides and lipids. To adopt these well-known metabolic phenomena, malignant cells need to reprogram transcription/splicing of many genes/transcripts, including those that code for some metabolic enzymes.

Pyruvate kinases are the crucial enzymes of the glycolytic pathway, which catalyze the last step of glycolysis. They transfer phosphate from phosphoenolpyruvate (PEP) to adenosine diphosphate (ADP), yielding adenosine triphosphate (ATP) and pyruvate. Two pyruvate kinases, PKM1 and PKM2, are coded by the *PKM* (Pyruvate Kinase M1/2) gene that is located at 15q23. Depending on mutually exclusive inclusion of exons 9 and 10 (both code for 56 amino acids) by splicing machinery, PKM1 and/or PKM2 will be expressed, respectively [2]. The exon 9 containing PKM1 isoform is the predominant form in normal adult cells. It exerts its pyruvate kinase activity in the cytoplasm, where it forms constitutively active tetramers. PKM1 presence in the nucleus was shown in several studies [3]. In highly proliferating fetal and cancer cells, PKM2 is the dominant isoform. This isoform is of the utmost importance for the adaptive nature of the Warburg effect. Under physiological conditions, the PKM2 isoform can be allosterically activated by fructose-1,6-bisphosphate (FBP) and non-essential amino acid serine, resulting in catalytically active PKM2 tetramers in the cytoplasm [4,5]. Nuclear localization of PKM2 can be a consequence of mutations present in exon 10 (H391Y, G415R, R399E) [6]. It can be also consequential to some post-translational modifications, such as phosphorylation of serine 37, which makes the PKM2 nuclear localization signal (NLS) more accessible to importin α5. In the nucleus, PKM2 regulates the activity of genes involved in glucose metabolism to promote the Warburg effect (reviewed in [7]). Phosphorylation of PKM2 has been particularly intensively studied in the field of molecular oncology, primarily as a consequence of commonly increased phosphotyrosine signaling. It was shown that phosphorylation of Y105 plays a critical role in decreasing the pyruvate kinase activity of PKM2, because it cannot be allosterically activated by FBP, nor it can form enzymatically active tetramers [8]. Similarly, formation of functional tetramers can also be inhibited as a consequence of oxidative stress, due to the oxidation of PKM2 cysteine 58 (C358) [9]. Fu et al. reported that hyperactivation of NRF2 (Nuclear Factor (Erythroid-Derived 2)-Like 2), a major transcription regulator of enzymes involved in antioxidative stress response, causes upregulation of Pkm2, its glycosylation and a dramatic increase in its tetrameric form in mice esophagus. High expression of Pkm2 tetramers was accompanied by overexpression of genes involved in glycolysis, pentose phosphate pathway (PPP) and glutathione (GSH) metabolism, which is highly dependent on serine [10]. In addition to its importance for maintaining cellular redox homeostasis, the serine biosynthetic pathway is an important alternative source of pyruvate in PKM1/PKM2-deficient cells. Simultaneous silencing of both isoforms in the mouse pancreatic cancer-derived cells (Kras^G12D/−^; p53^−/−^) did not affect the level of pyruvate nor did it impact their proliferative potential. Maintaining pyruvate level was shown to be highly dependent on alternative sources, among which the serine biosynthesis pathway was the most prominent one. It was shown to be dependent on the activity of phosphoglycerate dehydrogenase (PHGDH) [11].

In autochthonous mouse models of melanoma and breast cancer, PHGDH expression was advantageous for tumor growth as its activity is mandatory for keeping serine level increased in a low exogenous serine condition [12]. Ye et al. have shown that in conditions of high serine demand, and consequent depletion of exogenous serine supply, the cancer cell no longer activates PKM2. Reduced PKM2 activity, in response to serine deprivation, results in an accumulation of glycolytic intermediate 3-phosphoglycerate (3-PG), and its diversion into serine biosynthesis [13]. As endogenously synthesized serine replenishes the serine pool, which is needed for proliferating cells, serine activates PKM2 and restores glycolytic flux. Upon serine starvation, activation of the general control nonderepressible 2-activating transcription factor 4 (GCN2-ATF4) pathway upregulates the expression of three enzymes involved in serine biosynthesis: PHGDH, phosphoserine aminotransferase 1 (PSAT1), and phosphoserine phosphatase (PSPH) [13]. A few years ago, De Nicola et al. have shown that NRF2 positively regulates the transcription of PHGDH and mitochondrial isoform of serine hydroxymethyltransferase (SHMT2), an enzyme that converts serine into glycine in small cell lung cancer cells. Profiling of 79 non-small cell lung carcinoma (NSCLC) cell lines demonstrated a great heterogeneity in levels of serine and glycine, as well as in the expression of PHGDH [14]. The mechanism by which NRF2 increases PHGDH and SHMT2 transcription was shown to be indirect, through NRF2-mediated increase of ATF4 and its binding to PHGDH and SHMT2 promoters [14]. An important aspect of the pro-proliferative effect of SHMT2 is generating methyl-tetrahydrofolate, an essential intermediate for purine biosynthesis, in the process of converting serine into glycine [15]. A very recent report showed that SHMT2 promoter contains a strong STAT3 binding site in its promoter, but the physical binding of STAT3 for this region was not shown [16]. STAT3 activity is closely related to PKM2 protein kinase activity, as PKM2 can also use the PEP as the phosphate donor for transferring the phosphate group on STAT3 (P-Y705) [17]. This event correlates with increased cellular proliferation. 

Pleiotropic, beneficial effects of curcumin are attributed to its various modes of action which affect numerous molecules and signaling pathways. Its direct binding to cysteine residues of various proteins, including metabolic enzymes (such as PHGDH and SHMT2) and STAT3, may be very important for alleviating the Warburg effect [18,19,20,21]. Siddiqui et al. have recently reported that curcumin downregulates PKM2 expression in cancer cells, consequently decreasing the Warburg effect. The PKM2 downregulation coincided with the inhibition of the mammalian target of rapamycin (mTOR) pathway and consequential downregulation of hypoxia-inducible factor 1-alpha HIF1α [22]. Previously, PKM2 transcription has been shown to be activated on PI3K/mTOR-mediated HIF1α induction [23]. Jiao et al. reported that curcumin inhibits the PI3K/mTOR signaling pathway in lung cancer [24]. However, although present in normal cells (mouse embryonic fibroblasts), p53 activation-dependent inhibition of mTOR may be corrupted in cancer cells [25]. Therefore, it is conceivable to expect that, despite curcumin’s common targets in various cell types/tissues, its measured net effects would be cell-type specific [20]. 

For all these reasons, we chose four different cancer cell lines (three of squamous cells origin and one of adenocarcinoma origin) and explored some molecular events, which may be important for the metabolic response to curcumin in a cell-type specific fashion.

## 2. Results

### 2.1. Cellular Response to Curcumin is Cell-Type Specific

**Figure 1:**
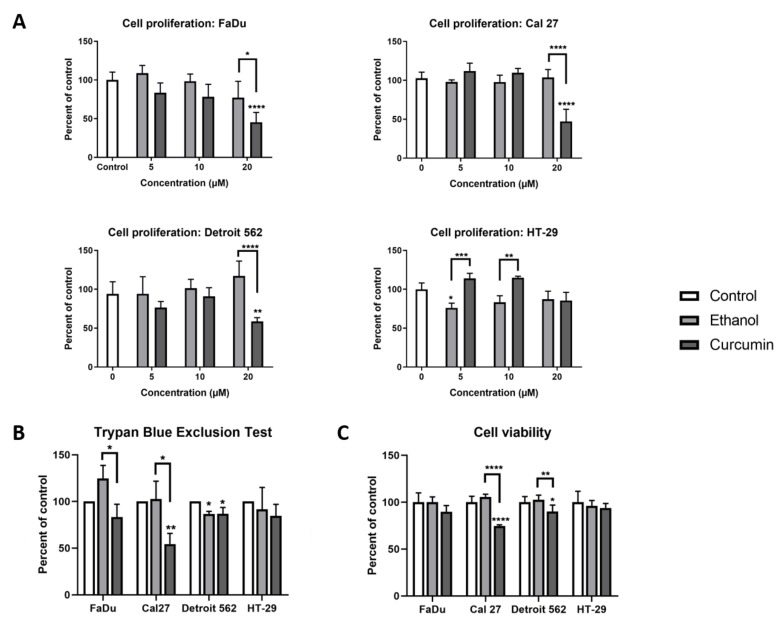
The effect of curcumin on cellular behaviour is cell-type specific. (**A**) Cell proliferation—BrDu assay with three different curcumin concentrations; (**B**) Trypan Blue Exclusion Test; (**C**) Cell Viability–MTT assay with 20 µM curcumin. One-way ANOVA with Tukey post-hoc test. * *p* < 0.05; ** *p* < 0.01; *** *p* < 0.001; **** *p* < 0.0001.

To determine curcumin’s effect on cellular biology, we first performed a BrdU (bromodeoxyuridine) assay, which was shown to be around 10 times more sensitive than a MTT (methylthiazolyldiphenyl-tetrazolium bromide) assay [26]. As shown on Figure 1A, strong anti-proliferative activity of curcumin was observed in planocellular, HNSCC (head and neck squamous cell carcinoma)-originating cell lines, when applied as 20 μM for 24 hours (Cal27 *p* < 0.001; FaDu *p* < 0.001; Detroit 562 *p* = 0.0061). This concentration of curcumin has induced mild anti-proliferative effect in adenocarcinoma-originating HT-29 cells, albeit without reaching a statistical significance, when compared with untreated cells. Only in this cell line were we able to record a mild pro-proliferative effect of curcumin with respect to ethanol treatment, when applied in a 10 µM concentration (*p* = 0.0017). Next, we performed cell counting by Trypan Blue Exclusion Test. As shown on Figure 1B, the strongest effect of curcumin was recorded in Cal27, with respect to both the untreated- (*p* = 0.0096) and ethanol treated cells (*p* = 0.0122). The effect was also observed in Detroit 562, although to a lesser extent (curcumin vs. control; *p* = 0.0212). In FaDu, the cytotoxic effect of curcumin was observed, but it reached statistical significance only with respect to ethanol treatment (ethanol vs curcumin: *p* = 0.0180), which itself had a pro-proliferative effect. The results of the MTT assay performed on the cells treated with 20 µM curcumin was complementary to the results obtained with the BrdU assay and Trypan Blue Exclusion Test (Figure 1C). Based on these findings, and our previously published results on HT-29 and Cal27 [27], it was reasonable to explore metabolic and molecular-genetic features of these cell lines after 24 hours of treatment with 20 µM curcumin.

### 2.2. Glucose Uptake and Lactate Production

The data related to glucose uptake and lactate production significantly varied among the treatments and should be considered at the observational level. Still, it is visible that the glucose uptake was influenced by ethanol. It seemed to be the least prominent in FaDu, in which curcumin treatment contributed to an additional decrease which was not accompanied with a decrease in lactate production. In HT-29, glucose uptake was also decreased by ethanol and even more so with curcumin. But, a slight increase in lactate production was observed in curcumin treatment. In Detroit 562 and Cal 27, ethanol treatment caused a decrease in glucose uptake but curcumin induced the opposite effect joined with an increase in lactate production. It reached statistical significance in Cal27 (ethanol vs. curcumin *p* = 0.0252). This set of data is presented as Supplementary Figure S1A,B.

**Figure 2:**
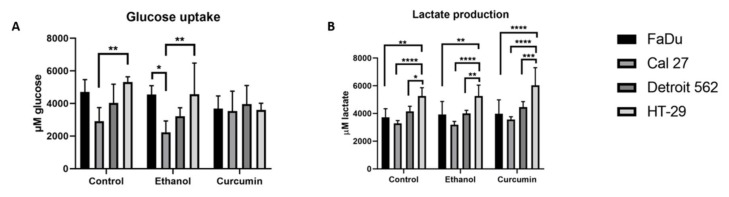
The difference in glucose uptake (**A**) and lactate production (**B**) among the untreated cell lines remains maintained through the treatments. A two-way ANOVA with Tuckey post-hoc test. * *p* < 0.05; ** *p* < 0.01; *** *p* < 0.001; **** *p* < 0.0001. All data was normalized to the results of a MTT assay.

We have further observed that the metabolic status of untreated cell lines differs with respect to both measured parameters. That encouraged us to compare them by a two-way ANOVA test which revealed the differences presented on Figure 2A,B. 

The difference in glucose uptake of control cells was the most prominent between Cal27 and HT-29 (*p* = 0.005). It remained significant in ethanol treated cells (*p* = 0.0024). Ethanol treatment also induced a significant difference in glucose uptake between FaDu and Cal27 (*p* = 0.0159). 

The level of lactate in the untreated cells was the lowest in Cal27, and highest in HT-29 (*p* < 0.0001). The differences remained the same during ethanol and curcumin treatments (*p* < 0.0001). The significant difference between HT-29 and other two HNSCC originating cell lines occurred in curcumin treatment (FaDu vs. HT-29; *p* < 0.0001, Detroit vs. HT-29; *p* = 0.0008). There were other, less significant differences, as shown on Figure 2B. 

In HT-29, we could not show any significant difference in glucose uptake with a one-way ANOVA (Supplementary Figure S1A). Only for that parameter, and only in this cell line, we performed an unpaired *t*-test for comparing the control and curcumin treated cells. That test has revealed a significant difference between these two groups (*p* = 0.0006) of cells. At the same time, it seems that lactate production remained the same; in curcumin treated, and in ethanol treated cells. This may suggest that the flux of glucose into the glycolytic pathway in HT-29 remains constant in these experimental conditions. It is quite possible that the utilization of glucose in serine biosynthetic pathway decreases. The results of RT-qPCR support this observation, since we have shown a decrease of expression in both, PHGDH and SHMT2 transcripts in curcumin treated HT-29 cells. 

It seems that the cell lines with the lowest glucose uptake (Cal27 and Detroit 562) in control conditions are the most sensitive, with respect to viability (Figure 2A), to curcumin treatment. The difference between Cal27 and HT-29 is also very obvious when considering lactate production. The significant difference present in a control condition remains preserved during both applied treatments. These results indicated that the strength of the Warburg effect differs among these cells and, in the present model, may be considered as an indicator of sensitivity to curcumin treatment.

### 2.3. Qualitative RT-PCR, RFLP and Amplicon Sequencing in Untreated Cells

**Figure 3:**
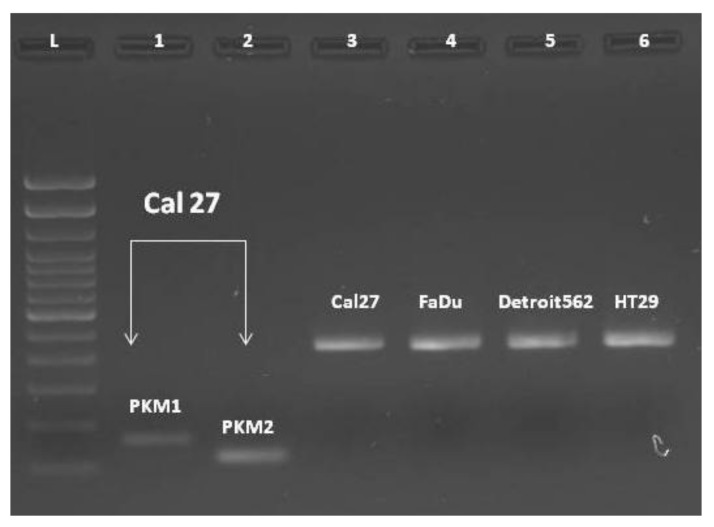
Presence of PKM1 and PKM2 isoforms in Cal 27; separately amplified (lines 1 and 2). Fragments obtained with PKM(C)F and PKM(C)R for obtaining the material for the RFLP analysis (lines 3–6). L: DNA ladder.

End-point PCR, with a combination of primers PKM1F/PKM1R and PKM2F/PKM2R in separate reactions, confirmed the presence of both isoforms, PKM1 and PKM2 (Figure 3, lines #1-#2). Then we combined the primers complementary to nucleotides in exons 7/8 and 11 (PKM(C) F and PKM(C) R) for obtaining the „mixed amplicon“, which was present as a band composed of 442 bp. (Figure 3, lines #3–6). We wondered about the proportion of PKM1 and PKM2 in this amplicon.

**Figure 4:**
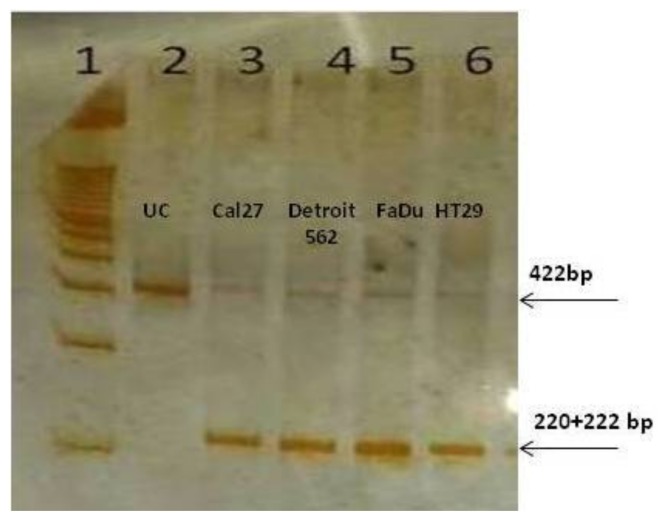
PKM2 transcripts prevail in the untreated cells. 1. DNA Ladder; 2. uncut fragment Cal27; 3–6. restricted fragments obtained with Kas1 in all four cell lines analyzed.

To our surprise, the end-point reaction with the primers common to both isoforms, generated only PKM2-amplicons in all cell lines, as confirmed by Restriction Fragment Length Polymorphism (RFLP) analysis with *Kas*1. This enzyme specifically cuts the sequence G↓GCGCC, which is present only in exon 10, yielding the bands of 220 and 222 bp (Figure 4). As presented, the amplicon was almost entirely cut, indicating the exclusive presence of the PKM2 isoforms in end-point PCR amplicons, in all four cell lines. The very weak signal at the position of 442 bp may correspond to the unrestricted PCR product, or it may still present the PKM1 isoform. 

**Figure 5:**
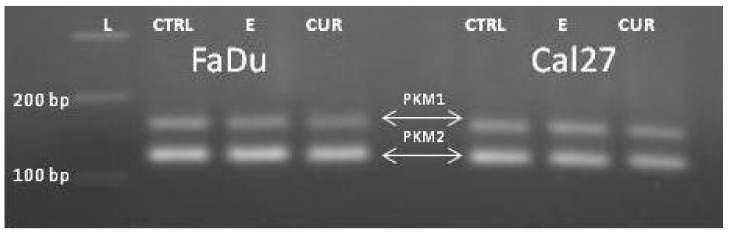
PKM2 isoform is the prevailing isoform in all cell lines (presented are FaDu and Cal27).

For the final confirmation of the prevailing isoform, we made a coamplification end-point PCR, with an equimolar concentration of PKM1 F and PKM2 F and an excess of PKM1R primer (Figure 5).

**Figure 6:**
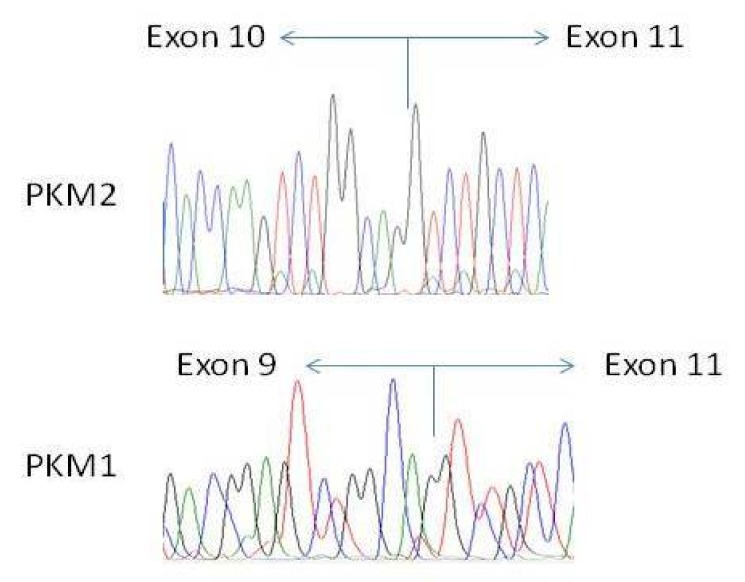
Sequencing of amplicons confirms the presence of both isoforms (exon 9 including–PKM1 and exon 10 including–PKM2), in all cell lines (presented is FaDu).

We have finally confirmed the authenticity of amplicons (Figure 3, lines #1-#2) and excluded the presence of mutations in PKM transcripts by sequencing both of them (Figure 6). 

Based on these data, we indirectly confirmed the activity of splicing machinery which favored inclusion of exon 10 and, at the same time, excluded the presence of mutations in both isoforms in the region covered by the primers used. We continued with precise quantification.

### 2.4. Quantitative PCR (RT-qPCR)

**Figure 7:**
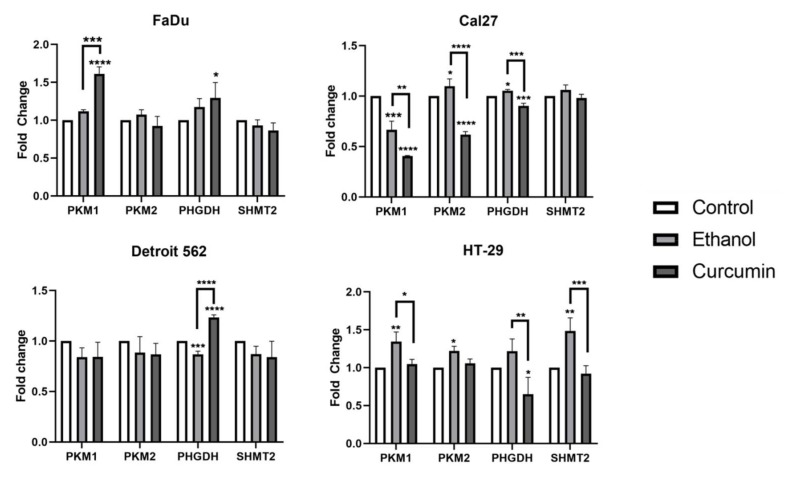
The effect of ethanol and curcumin on transcription of PKM1, PKM2, PHGDH and SHMT2 in four different cancer cell lines. One-way ANOVA with Tukey post-hoc test. * *p* < 0.05; ** *p* < 0.01; *** *p* < 0.001; **** *p* < 0.0001.

We wanted to explore a potential effect of ethanol/curcumin on the expression of transcript variants PKM1 and PKM2. We also performed transcriptional analyses of PHGDH and SHMT2, the genes whose protein products play an important role in serine metabolism. Quantification of target transcripts was performed with two commonly used housekeeping genes, GAPDH and TBP, using TaqMan chemistry. With respect to the solvent, we were able to show the effect of ethanol which significantly varied among the cell lines (Figure 7). For example, it has induced a significant increase of PKM1, PKM2 and SHMT2 transcripts in HT-29, the cell line that was shown to be the most resistant to curcumin (control vs. ethanol: PKM1 *p* = 0.0021; PKM2 *p* = 0.0311; SHMT2 *p* = 0.0016). The decrease induced by curcumin was significant for PKM1 (ethanol vs. curcumin: P= 0.0134), PHGDH (ethanol vs. curcumin: *p* = 0.0029), and SHMT2 (ethanol vs. curcumin: *p* = 0.0006) but, at the end, the level of all transcripts, except PHGDH in HT-29 exposed to curcumin did not differ with respect to the control (control vs. curcumin: PHGDH *p* = 0.0373). 

Stimulatory effect of ethanol on SHMT2 transcription was absent in HNSCC-originating cell lines. In Cal27, stimulatory effect of ethanol on PKM2 and PHGDH transcription (control vs. ethanol: PKM1 *p* = 0.0448; PHGDH *p* = 0.0195) was abolished by curcumin (ethanol vs. curcumin: PKM2 *p* < 0.0001; PHGDH *p* = 0.0001). A significant decrease was also recorded with respect to the untreated cells (control vs. curcumin: PKM2 < 0.0001; PHGDH = 0.0006). Cal27, which was shown to be the most sensitive with previously performed cellular tests, was the only model in which both ethanol and curcumin treatments induce decrease of PKM1 transcript (control vs. ethanol: PKM1 *p* = 0.0002; control vs. curcumin PKM1 *p* < 0.0001). Thus, the silencing effect of curcumin on PKM1 and PKM2 transcripts is significant and is the most prominent in Cal27 cell line.

The only significant change in Detroit 562 was related to the PHGDH transcripts which were slightly decreased in ethanol treated cells (control vs. ethanol: *p* = 0.009) and then strongly increased by curcumin treatment (control vs. curcumin: *p* < 0.0001). Transcription of PHGDH was also slightly increased in FaDu treated with curcumin (control vs. curcumin: *p* =0.0253). Curcumin treated FaDu was the only cell line in which the level of PKM1 transcripts increased with respect to the control and ethanol treatment (control vs. curcumin: <0.0001; ethanol vs. curcumin *p* =0.002).

The effect of ethanol is clearly seen in all cell lines. For example, when comparing curcumin treated cells with the control, untreated cells, without having ethanol treatments included, one may conclude that curcumin does not influence transcription of PKM1 and PKM2 in HT-29. Obviously, the silencing effect was there, but is “covered” by activating effect induced by ethanol itself. The ethanol also influenced transcription of house-keeping genes, but not in all cell lines: The Ct values obtained by TaqMan GAPDH and TBP was stable in Cal27, but highly unstable in Detroit 562 and HT-29. For example, the mean value of these house-keepers obtained on two HT-29 templates and eight measurement resulted with GAPDH and TBP Ct values of 19.1094, 20.2659 and 18.1977 (GAPDH) and 27.4840, 26.9286 and 27.8244 (TBP) in control, ethanol treated and curcumin treated samples. The accurate quantification was impossible. For that reason, we needed to test some other housekeepers (Table 1), and we did so by SYBR^®^ Green chemistry. Among all of them, only beta actin, hydroxymethylbilane synthase (HMBS)and peptidyl-prolyl isomerase A (PPIA) were shown to be highly stable, in all cell lines (for example: Detroit 562: HMBS: Ct values: 23.7625 (control), 23.5771 (ethanol), 23.8194 (curcumin)). We also compared our TaqMan (GAPDH) data with the SYBR^®^ Green (beta-actin) data using Cal27. The results, expressed as a fold change were almost identical.

This data clearly points out that the effect of ethanol as a solvent must be seriously considered when performing experiments of this type. 

### 2.5. Western Blot

**Figure 8:**
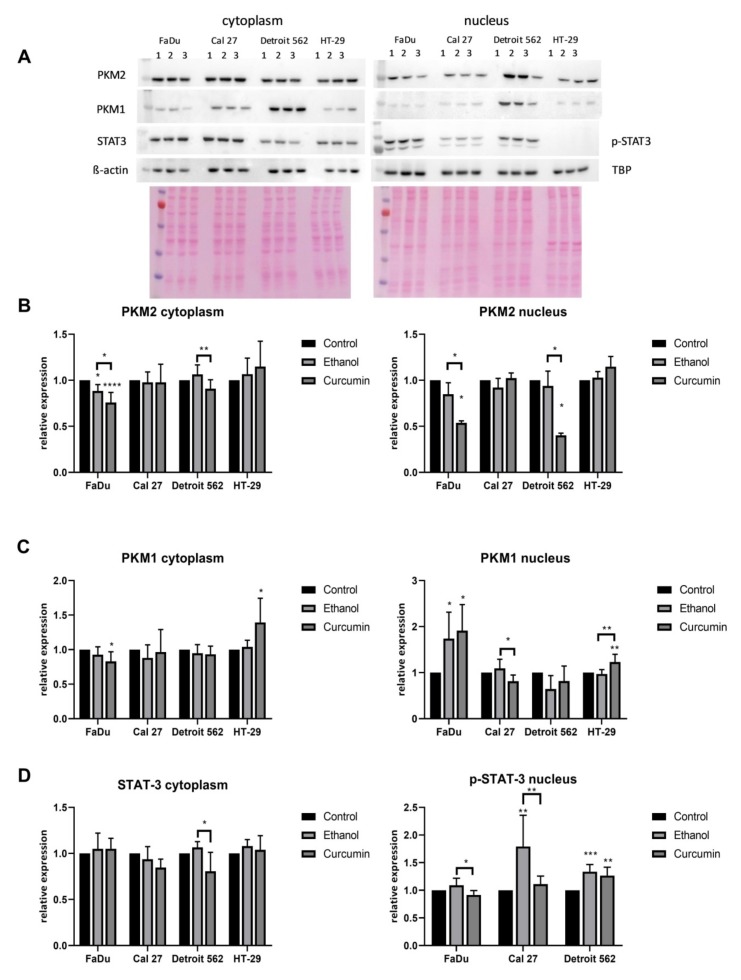
Expression of PKM1, PKM2 and STAT3 in cancer cell lines. Representative Western blots of cytoplasmic and nuclear PKM1, PKM2 and STAT3 content under different: (**A**) Western Blot. Lines: #1: control; #2: ethanol; #3: curcumin. Relative expression is calculated as compared to the untreated cells. (**B**)-cytoplasmic and nuclear PKM2; (**C**)-cytoplasmic and nuclear PKM1; (**D**)-cytoplasmic non-phosphorylated and nuclear phosphorylated STAT3 (Y705). One-way ANOVA with Tukey post hoc test was used to test the differences in relative expression of selected proteins based on minimally five independent experiments. Values are shown as mean ± SD. *n* = 5–7. * *p* <0.05; ** *p* < 0.01; *** *p* <0.001; **** *p* <0.0001.

We have also performed the Western blot (Figure 8). PKM1 and PKM2 were analyzed in both cellular fractions. Unphosphorylated STAT3 was analyzed in the cytoplasmic fraction, while the activated P-STAT3 (Y705) was analyzed only in the nuclear fraction.

What is clearly visible is the absence of the P- STAT3 (Y705) in the nuclei of HT-29 cells. The analysis was repeated three times, and the result was always the same. This phenomenon was described and explained by Corvinus at al., who showed that some colon cancer derived cell lines (HT-29 was included), contrary to the colon cancer tissues, do not express the active form of STAT3, unless incubated with IL-6 [28]. Curcumin, having the strong anti-inflammatory effect, could not influence this biological mechanism. 

The protein level of PKM1 in Detroit 562 was not significantly affected by applied treatments. The significant, and the opposite change of the PKM1 transcript level in FaDu and Cal27 (increase in FaDu, decrease in Cal27) induced by curcumin treatment was followed with the similar changes of the PKM1 protein, but only when considering the nuclear fraction (control vs. curcumin FaDu *p* = 0.0104; Cal27 *p* = 0.0126). In curcumin treated HT-29, there was a significant increase of the PKM1 protein in the nuclear fraction (control vs. curcumin *p* = 0.0073; ethanol vs. curcumin P=0.0029), and an increasing trend in the cytoplasmic fraction, although the RT-qPCR indicated its strong increase only in the ethanol treated cells. 

Although we did not measure a significant change of the PKM2 transcript in Detroit 562 and FaDu cells, there was a significant curcumin-associated decrease of PKM2 protein in the nuclear and both cellular fractions, respectively (control vs. curcumin; Detroit 562: *p* =0.0161; FaDu (nucleus): *p* = 0.0159, FaDu (cytoplasm): *p* < 0.0001). In the cytoplasm of Detroit 562, the difference was statistically significant only when comparing ethanol- and curcumin treated cells (*p* = 0.006). The strong decrease of PKM2 transcripts which was present in curcumin treated Cal27 cells was not obvious at the protein level. In HT-29, a mild but significant increase of PKM2 transcript in ethanol treated cells was not confirmed at the protein level, which remained the same in both cellular fractions. 

It seems that in the nucleus of the curcumin treated FaDu cells PKM1 compensates for the loss of PKM2, which may influence their rate of proliferation (a decrease). The same mechanism does not seem to be present in curcumin treated Cal27 cells (decreased and unchanged nuclear PKM1 and PKM2, respectively). With respect to curcumin sensitivity, which significantly differs between Cal27 and HT-29, it remains to be explored whether the PKM1 decrease contributes to Cal27 vulnerability, and whether simultaneous increase of PKM1 (and possibly PKM2) contributes to HT-29 resistance.

With respect to unphosphorylated STAT3, the strong cytoplasmic signal was present in all cells. Only in Detroit 562, there was a significant decrease of STAT3 in the cytoplasm, when comparing ethanol- and curcumin treated cells (*p* = 0.0350). The stimulatory effect of ethanol on STAT3 activation is well-visible in the nuclei of all three cell lines. Although curcumin treatment significantly decreases the amount of activated STAT3 (ethanol vs. curcumin: FaDu *p* = 0.0130; Cal27: *p* = 0.0041; control vs. curcumin: Detroit 562: *p* = 0.0041). This means the level of the P-STAT3 only in Detroit 562 remains statistically significantly higher than in the corresponding untreated cells. Only in curcumin treated FaDu, the signal of the P-STAT3 is below the strength of the signal of untreated cells, but it does not reach statistical significance.

## 3. Discussion

Our starting hypothesis was that curcumin modifies the metabolic status of the cell in a cell type-specific fashion. Siddiqui et al. have recently shown that curcumin dissolved in DMSO negatively influences the Warburg effect through mTOR-HIF1α-mediated silencing of PKM2, in four cell lines originating from four different epithelial malignant tumors (lung, cervix, prostate and breast). They showed that a decrease of PKM2 (mediated by curcumin or by targeted PKM2 silencing) significantly reduces aerobic glycolysis and is also consequential for cell survival. Curcumin’s influence on PKM1 was not addressed, and the effect was not shown as cell-type specific [22]. We wanted to explore the effect of curcumin on both isoforms in four different cancer - originating cell lines. Special attention in this current research was dedicated to exploring the potential effect of curcumin and the solvent, absolute ethanol, which was shown as a strong modifier of the results of the MTT assays [29]. Dimethyl sulfoxide (DMSO), a commonly used solvent for curcumin, also was recently shown to be a strong modifier of cellular epigenome and, consequentially, a transcriptome in cardiomyocytes and hepatocytes [30]. The ethanol effect was obvious in all experiments. It was particularly obvious with respect to P-STAT3. Ethanol stimulates STAT3 activation and curcumin, acting in the opposite direction, brings it back to the level of an untreated cell. Thus, when comparing only untreated and curcumin treated cells, without testing the solvent, one could wrongly conclude that curcumin has no effect on STAT3 activation.

At the level of transcripts, the opposing effects of ethanol and curcumin were obvious and reached statistical significance for PKM1 and SHMT2 in HT29, for PKM2 in Cal27, and for PHGDH in all cell lines but FaDu. Clearly, the cellular response was different. It is hard to estimate how these changes associated with corresponding proteins when considering their total amount, as we measured PKM1 and PKM2 in separate cellular fractions. Still, we were able to observe the significant opposing effects of ethanol and curcumin on PKM1 in the nuclear fraction of Cal27 and cytoplasmic fraction of PKM2 in Detroit 562. All these point out the necessity for careful exploration of the solvent when various compounds are tested.

Quite rarely, in vitro experiments compare curcumin and its solvent. In that case, the status of biological targets in untreated cells remain entirely unknown. Far more often, one can find a comparison of untreated vs. treated curcumin cells. In that situation, the potential effect of the solvent is entirely omitted. That may be one of reasons that numerous data coming from various research groups cannot be compared–as the models of investigation may look similar but are, as shown here, entirely different. The comprehensive data on the strong DMSO effect on the two types of untransformed cell types was published only last year [30]. We were not able to find similar data with respect to ethanol application. Of the greatest importance, while keeping in mind that researchers dissolve curcumin in either DMSO or ethanol, one can really ask how comparable the data obtained are. We did not perform experiments with DMSO-solved and ethanol-solved curcumin and compare the data set. However, in our view, that should be done soonest.

The cell lines used in this research varied with respect to glucose uptake and lactate production in basal (untreated) condition. The most prominent difference existed between Cal27 and HT-29 and also applied to their response to ethanol and curcumin. The most sensitive cell line, Cal27, had the lowest glucose uptake and the lowest rate of lactate production. The most resistant cell line, HT-29, had the highest glucose uptake and the highest rate of lactate production. The most prominent difference on the molecular level (besides the one related to STAT3 activation as already explained), was the status of the nuclear PKM1, which decreased in curcumin treated Cal27, while increasing in HT-29. With respect to the control Cal27 cells, it was obvious that curcumin treatment decreased the transcriptional level not only of PKM2, but also of PKM1. Thus, we cannot exclude the possibility that curcumin has a negative influence on the PKM promoter only in Cal27.

As shown by Prakasam, cancer cells express both PKM isoforms which interact to generate heterotetrameric cross-oligomers and jointly contribute to the overall pyruvate kinase activity in a cancer cell. They also showed that both isoforms, PKM1 and PKM2, drive the glycolysis to yield ATP which indicates “that PKM1 in cancer cells is not just a bystander” [3]. The importance of PKM1 in the tumorigenesis of neuroendocrine tumors, including small-cell lung cancer (SCLC), was recently shown by Morita et al, who observed the necessity of PKM1 expression for SCLC cell proliferation [31]. Indeed, in Cal27, curcumin treatment did not affect the level of PKM2 protein, but it did decrease the level of nuclear PKM1. This may be one of the reasons for the obvious sensitivity of Cal27 to curcumin. On the other hand, the most resistant cell line, HT-29, responded to curcumin with a significant increase of PKM1 isoform and probable increase of the PKM2 isoform. Our results are not the first to demonstrate the insensitivity of HT-29 to curcumin [32].

There is also data showing that loss of PKM2 leads to compensatory expression of PKM1 in the tissues that normally express PKM2 [33]. 

This phenomenon was observed in the nuclear but not cytoplasmic fraction of curcumin treated FaDu cells, but not in the other cell lines.

It is surprising to see that curcumin balances the glucose uptake in all cell lines, which makes them quite similar with respect to that parameter. However, the differences in lactate production among the cell lines remain the same. This indicates that the glucose utilization used for lactate production does not significantly change. This may be an indication that the use of glucose in PPP and serine biosynthesis pathway decreases. This seems to be especially relevant for HT-29. We have further examined curcumin’s effect on the transcriptional status of 3-phosphoglycerate dehydrogenase (PHGDH), and serine hydroxymethyltransferase 2 (SHMT2). *In situ* proteomic profiling of curcumin targets in a HCT116 colon cancer cell line reported 197 curcumin-binding proteins, including PKM1, PKM2 and SHMT2 [34]. We also proposed a model for curcumin’s binding to SHMT2 [20]. As already stated in the Introduction, PHGDH was shown as curcumin’s target in Angelo’s study [19]. Curcumin was also shown to directly interact with the Cysteine 259 residue of STAT3 and induce apoptosis in H-Ras transformed human mammary epithelial cells [21]. Other than these studies, there is no data with respect to curcumin’s action on these biomolecules at the level of their mRNA and corresponding protein. We have shown that curcumin exerts the effect, which was a significant decrease of SHMT2 in HT-29. It is quite interesting that PHGDH transcript decreased in Cal27 and HT-29 which were, with respect to curcumin sensitivity, significantly different, while it increased in FaDu and Detroit 562. These findings are certainly important, since the changes, if present at the protein level, may significantly influence the Warburg effect.

In non-stimulated cells, STAT3 is kept in an inactive cytoplasmic form. When activated through phosphorylation on a critical tyrosine residue (Tyr 705), STAT3 translocates into the nucleus where it behaves as a transcription activator for a broad array of targeted genes [35]. Gao et al have shown that PKM2 kinase also phosphorylates STAT3 at Y705 [17]. 

Only in FaDu, did we observe a decrease of P-STAT3 associated with a decrease of PKM2. An increased level of P-STAT3 in Detroit 562 turned out to be a consequence of ethanol’s action, which remained unchanged in curcumin treated cells. That scenario was not present in Cal27. Instead, it appeared that ethanol itself strongly contributes to an increase of P-STAT 3, which may or may not be associated with PKM2 kinase activity (of note, the level of PKM2 in the nucleus did not change). In Detroit 562, it appeared that PKM2 has no effect on STAT3 phosphorylation.

In this research, we have shown that the basic cellular background relevant for metabolic adaptation, including the Warburg effect, significantly differed among four cancer cell lines. These differences are also responsible for cellular response to any kind of intervention. This is very relevant to human medicine with respect to personalized treatments. We are aware that the parameters on which we followed-up make only a very small part of, yet incompletely understood, complicated regulatory network that is shaping the glycolytic metabolic pathway in cancer cells. However, we hope that this research shows how important and sensitive cellular background can be when responding to a strong pleiotropic molecule.

## 4. Materials and Methods

### 4.1. Cell lines and Cell Culture Conditions

Four cancer originating human cell lines were used in this research. Three cell lines were of head and neck squamous cell cancer (HNSCC) origin: 1. Detroit 562 (pleural effusion of metastatic pharyngeal cancer); 2. Cal 27 (tongue carcinoma), and 3. FaDu (hypopharyngeal cancer). The fourth cell line, HT29, originates from the colorectal adenocarcinoma. Detroit 562 and HT-29 were purchased from Sigma-Aldrich (St. Louis, MO, USA), while the other two cell lines were obtained from the American Type Culture Collection (ATCC, LGC Standards GmbH, Wesel, Germany). The curcumin was purchased from Sigma-Aldrich (C1386; St. Louis, MO, USA) and dissolved in absolute ethanol; the curcumin stock solution was 10 mM.

The cells were cultured in T75 cell culture flasks (Sarstaedt AG&Co.KG, Nümbrecht, Germany), in Dulbecco’s Modified Eagle’s Medium (DMEM, D5796; Sigma-Aldrich, St. Louis, MO, USA), with addition of 10% fetal bovine serum (FBS, Sigma-Aldrich, St. Louis, MO, USA), without antibiotics, in humidified atmosphere at 37 °C and in the presence of 5% CO_2_.

Before the experiments, cells were detached from the surface with 0.25% (*w*/*v*) Trypsin-0.53 mM EDTA (Ethylenediaminetetraacetic acid; Sigma-Aldrich, St. Louis, MO, USA) solution, and counted with the Trypan Blue Exclusion Test on Bürker–Türk hemocytometer (Brand, Wertheim, Germany). 

The concentration of curcumin used in experiments performed for estimating the metabolic status of the cells was estimated from data related to cell proliferation and viability (BrdU and MTT assays) after exposure to various curcumin concentrations. Based on these two assays we have chosen 20 µM concentration of curcumin, which exerted considerable inhibitory effects on proliferation, without inducing the high level of MTT recordable cytotoxicity, in 96-well plates. This concentration is the same as the curcumin concentration that was used by other authors studying the effects of curcumin on cancer cell metabolism [22]. As a “solvent control” reactions, the final concentration of ethanol was equal to the final ethanol concentration (0.2%) added from curcumin solution. 

### 4.2. Cellular Viability and Proliferation; Additional Counting of Treated Cells

For performing assays on cellular viability and cellular proliferation, 1 × 10^4^ cells were seeded in 96-well plate (TPP, Trasadingen, Switzerland), in 100 µL DMEM supplemented with a 10% FBS. After 24 hours, which was the time needed for attachment of cells to the surface, cells were treated for 24 hours with the following curcumin concentrations: 5, 10, 15 and 20 µM and corresponding concentration of ethanol (solvent control), respectively. Cellular viability was assayed with EZ4U assay (Biomedica, Vienna, Austria) according to manufacturer’s instructions. Briefly, 20 µL of the dye substrate (tetrazolium salt) was added to each well. After a two hours incubation, absorbance was measured at 450 nm wavelength, with 620 nm as reference, using the microplate reader Multiskan EX (Thermo Electron Corporation, Shanghai, China). Cell proliferation was measured using the Cell Proliferation ELISA, BrdU (colorimetric) Kit (Roche Applied Science, Mannheim, Germany). In brief, after 24-hours of treatments, BrdU solution was added to each well and removed after two hours, which is a time-window needed for BrdU incorporation into the DNA of proliferating cells. Appropriate controls were included, as suggested by the manufacturer. ELISA measurement of the incorporated BrdU was performed according to the manufacturer’s instructions: the absorbance of reaction product TMB (3,3′,5,5′- tetramethyl-benzidine diimine) was measured at 450 and 620 nm as a reference wavelength using the microplate reader Multiskan EX.

Considering that the cellular mechanism of MTT reduction into formazan likely involves reaction with NADH or similar reducing molecules that transfer electrons to MTT [26]–thus, it is dependent on cellular metabolism which may significantly vary among different cell lines [36], additional confirmation of results obtained by cellular viability and proliferation assays was performed by cell counting after the treatment with already chosen curcumin concentration. 1x10^6^ cells were seeded in T25 cell culture flasks (Sarstedt AG&Co.KG, Nümbrecht, Germany), in 5 mL of DMEM supplemented with 10% FBS. After growing for 24 hours, cells were treated with curcumin or equivalent concentration of ethanol for 24 hours and harvested with 0.25% (*w*/*v*) Trypsin-0.53 mM EDTA solution and counted with the Trypan Blue Exclusion Test in Bürker-Türk hemocytometer. 

### 4.3. Glucose Uptake and Production of Lactate 

Cells were cultivated in 96-well plates (TPP, Trasadingen, Switzerland) and treated with ethanol and 20 µM curcumin, as described previously. 

Glucose uptake and lactate production were measured in media with Glucose-GloTM and Lactate-GloTM assays (Promega, Madison, WI, USA), respectively, according to manufacturer’s instructions. Briefly, the medium taken from the each well was diluted 100x for glucose assay and 50x for lactate assay, placed in a white 96-well plate (Thermo Fisher Scientific, Nunc A/S, Roskilde, Denmark) and mixed for 60 seconds with an equal volume of Glucose detection reagent and Lactate detection reagent, respectively. After one hour incubation, luminescence was recorded on a plate reader Infinite 200 PRO (Tecan Group Ltd., Männedorf, Switzerland). Lactate concentration was interpolated from a standard curve ranging from 0 to 200 µM. Glucose uptake was calculated by interpolation of the difference between control culture media without cells and samples media, from a standard curve ranging from 0 to 50 µM. 

The concentrations obtained were corrected with respect to the results of cell viability assay.

### 4.4. RNA Extraction and Reverse Transcription (RT)

The total RNA was extracted from the cells cultivated and treated in 25 cm^2^ flasks (Sarstedt AG&Co.KG, Nümbrecht, Germany). After removal of the medium, the extraction of total RNA was performed by TRIzol (Invitrogen, Carlsbad, CA, USA), according to the manufacturer’s instructions. Agarose gel (1%)/ethidium bromide (EtdBr) (Sigma-Aldrich, St. Louis, MO, USA) electrophoresis was performed for determination of RNA integrity. all samples were further purified with gDNA Removal Kit (Jena Bioscience, Jena, Germany), according to the manufacturer’s instructions. The final concentration and purity of extracted and purified RNA was determined spectrophotometrically (BioSpec-nano, Shimadzu Biotech, Japan) by measuring the absorbance at: 230, 260 and 280 nm. The samples were stored at −80 °C.

A High-Capacity cDNA Reverse Transcription Kit (Thermo Fisher Scientific, Waltham, MA, USA), with anchored Oligo(dT)_23_ primers (Sigma-Aldrich, St. Louis, MO, USA) was used for synthesis of cDNA from 1 µg of total RNA in a 20 μL volume, according to the manufacturer’s instructions. The reaction conditions were: 25 °C/10 min; 37 °C/120 min; 85 °C/5 min; 4 °C/indefinite. After finalization of the reverse transcription reaction, 80 μL of sterile, deionized water was added to the tubes for achieving a total volume of 100 μL of cDNA, which was used for subsequent reactions.

### 4.5. End-Point PCR and Amplicon Sequencing

All primers used in this research are listed in the Table 1.

The efficacy of reverse transcription was assessed with the end-point polymerase chain reaction (PCR) using the primer pair GAPDH 1/GAPDH 2. For discovering the potentially present traces of contaminating DNA, the primer pair GAPDH2/GAPDH3 was used, as the sequence of the GAPDH3 primer is complementary to the nucleotides in intron 5 [37]. One microliter of diluted cDNA was used as a standard volume in all end-point PCR reactions. The polymerase chain reaction was carried out in GeneAmp PCR System 2400 (Applied Biosystems, Foster City, CA, USA). The reaction mixture (12.5 μL) contained AmpliTaq 360 Gold Master Mix and GC Enhancer (Thermo Fisher Scientific, Waltham, MA, USA), home-made nuclease free-water and primers (final concentration: 400 nM).

For simultaneous detection of PKM1 and PKM2 transcripts by RT-PCR, two forward primers PKM1 F and PKM2 F, complementary to nucleotide sequence in exon 9 (NM_182471.4) and 10 (NM_002654.6), respectively, were constructed. The reverse primer used was complementary to nucleotide sequence in exon 11 (PKM(C)R), which is common to the both transcriptional isoforms. The expected sizes of amplicons were 157 bp and 118 bp, for PKM1 and PKM2, respectively. We expected that this combination of primer sequences would allow for a clear distinction of the PKM1 and PKM2 transcripts.

After successful separation of PCR products in 2% agarose gel, two bands of different size were cut out of the gel and purified using a GenElute Gel Extraction Kit (Sigma-Aldrich, St. Louis, MO, USA). The quality and quantity of the final column eluate was loaded in 2% agarose gel stained with EtdBr in order to determine the purity and amount of template for the sequencing reaction. The total volume of 13 µL sequencing reaction contained approximately 50 ng of the purified template per 100 bps, 1 µL of the reverse primer (primer concentration 3,2 pmol/µL) used in PCR, and home-made sterile, deionized water. The samples were sequenced at the DNA Sequencing Core Facility of the Rudjer Boskovic Institute. 

For performing the end-point PCR which does not allow for discrimination of two isoforms based on their size, but based on their sequence restriction sites, another pair of primers was constructed (Table 1): PKM(C)F and PKM(C)R. With this primers pair, the expected size of the amplicon was 442 bp. Since exon 10, contrary to exon 9, contains a unique restriction site for endonuclease KasI (G↓GCGCC) exposure of the amplicon to this enzyme in the RFLP (Restriction Fragment Length Polymorphism) procedure would generate two fragments originating from PKM2 (220 + 222 bp), but not PKM1. Briefly, 10 U of KasI (New England BioLabs, Ipswich, MA, USA) was added to 10 µL of amplicon, appropriate buffer and water in a final volume of 25 µL. After four hours of incubation at 37 °C, 10 µL of the reaction product was loaded on nondenaturing 10% PAGE and silver-stained, as described [38].

### 4.6. Real Time RT-qPCR

For quantification of all transcripts (PKM1, PKM2, PHGDH, SHMT2), we used TaqMan chemistry. The reactions were performed in a 7300 Real-Time PCR System in The Microamp 96-well rxn plates, (Applied Biosystems, USA), in triplicate for each template and for each probe. We worked with at least three biological replicates, which we tried to associate with three consecutive passages. The reaction mix consisted of 1.5 μL of cDNA template, 10 μL of TaqMan Fast Advanced Master Mix (Thermo Fisher Scientific), 1 μL of the probe and 7.5 μL of sterile, deionized water. The amplification conditions were: incubation 50 °C/2 min + 95 °C/10 minutes, followed by 40 cycles; 95 °C/15 sec, 60 °C/60 sec. The reactions were performed with two house-keepers: GAPDH-Hs99999905_m1 (as we used GAPDH in our end-point PCR reactions) and TBP - Hs00920495_m1 (as we used the TBP in our WBs). The sets of primers and probes for targets were: Hs00987255_m1 (PKM1); Hs00987262_g1 (PKM2); Hs01059260_g1 (SHMT2) and Hs00198333_m1 (PHGDH). 

The stability of other eight housekeeping genes was tested with SYBR^®^ Green. We have constructed primers for amplifying the fragments shorter than 200 bp, as shown in Table 1. For quantifying PKM1 and PKM2, we used PKM1F, PKM2F and PKM(C)R primers. We finally constructed the new PHGDH and SHMT2 primers for obtaining the amplicons of 107 and 169 bps, respectively. 

The reaction mix contained 6 µL of sterile deionized water, 2 µL of mixed forward and reverse primers (5 µM each), 10 µL of PowerUp SYBR^®^ Green Master Mix and 2 µL of target template.

The amplification protocol was: incubations 50 °C + 95 °C, each for 2 min, followed by 40 cycles; 95 °C/15 sec, 58 °C/15 sec, 72 °C/60 sec.

### 4.7. Protein Extraction and Western Blot Analyses

The cells were cultured for 48 hours at a density of 1 × 10^6^ in T25 flasks (Sarstedt AG&Co.KG, Nümbrecht, Germany), in 5 mL of DMEM- high glucose (DMEM, D5796; Sigma-Aldrich, St. Louis, USA) supplemented by 10% FBS (Sigma-Aldrich, St. Louis, USA). Proteins were extracted with NE-PER nuclear and cytoplasmic extraction reagents (Thermo Scientific -Pierce Biotechnology, Rockford, USA), with addition of protease inhibitor (Complete Mini Protease Inhibitor Cocktail Tablets; Roche Applied Science, Mannheim, Germany). The amount of protein was determined by the Bradford method [39]. Absorbance was measured at 620 nm using the microplate reader Multiskan EX (Thermo Electron Corporation, Shanghai, China). After mixing with Laemmli buffer and boiling for 5 min at 95 °C, equal amounts of proteins (4–10 μg) was loaded on the denaturing PAGE gel (9% resolving and 5% stacking), separated electrophoretically and transferred to nitrocellulose membranes (Roti®-NC, Carl Roth, Karlsruhe, Germany). The membranes were stained with Ponceau S solution (Sigma Aldrich, St. Louis, USA) and scanned for estimating the efficiency of the transfer. After incubating membranes with 5% nonfat milk (Cell Signaling Technology, Danvers, USA) in Tris-buffered saline (TBS; 50 mM Tris-Cl, 150 mM NaCl, pH 7.6) containing 0.1% Tween-20 for 1 h, the membranes were probed overnight with the following primary antibodies (all rabbit monoclonal, but anti-SHMT2 (rabbit polyclonal); Cell Signaling Technology, Danvers, USA): anti-PKM1 (1:1000; CST: #7067); anti-PKM2 (1:1000; CST:#4053); anti-PHGDH (1:1000; CST:#66350); anti-SHMT2 (1:1000; CST:#12762); anti-STAT3 (1:1000; #12640); anti phospho-STAT3 (Tyr705) (1:2000; CST:#9145); anti-TBP (1:2000; CST:#44059); anti-β-actin (1:1000, CST:#8457). The last two antibodies were used as the loading controls for nuclear and cytoplasmic fractions, respectively. The expected molecular weights of detected proteins were: PKM1 and PKM2- 60 kDa; STAT3 and p-STAT3–79 and 86 kDa (STAT3α and STAT3 β); TBP 35-45 kDa; β-actin 45 kDa. Membranes were washed for three times with TBST (0.1% Tween 20 in 1x TBS) and the immunoreactive bands were detected with an HRP- linked anti-rabbit IgG secondary antibody (1:2000; CST: #7074). Immunological complexes were visualized using SuperSignal^™^ West Pico PLUS Chemiluminescent Substrate (Thermo Scientific, Rockford, USA) and Uvitec Alliance Q9 Mini (UVITEC, Cambridge, UK). Protein expression levels were quantified using Nine Alliance analysis software. The relative change of signals obtained was calculated after normalization according to the loading controls and Ponceau S signals.

### 4.8. Statistical Analyses

Each experiment related to cellular biology (viability, proliferation) was performed in technical triplicates or quadriplicates and repeated minimally three times. The data obtained was analyzed with a one-way ANOVA and Tukey post-hoc test, as indicated in the figure legends. The same principle was applied for producing and analyzing the data obtained with molecular biology methods, in biological triplicates and technical multiplicates. For both analyses and visualization, GraphPad 6.0 was used. Statistical significance of differences obtained for all data analyzed was considered significant at *p* < 0.05.

## Figures and Tables

**Table 1:** Primers used in end-point PCR and quantitative PCR based on SYBR^®^ Green chemistry.

Primers	Primer Sequences	Amplicon Size
	**GAPDH Primers**	
GAPDH1	5′AACGGATTTGGTCGTATTGGGC3′	600 bps
GAPDH2	5′AGGGATGATGTTCTGGAGAGCC3′	
GAPDH2	5′AGGGATGATGTTCTGGAGAGCC3′	644 bps
GAPDH3	5′AAGCTGACTCAGCCCGCAAAGG3′	
	**PKM Primers**	
PKM1 F	5′TCCACAGACCTCATGGAAGC3′	157 bps
PKM1 R	5′GATTCCGGGTCACAGCAATG3′	
PKM2 F	5′GAGGCCTCCTTCAAGTGCTG3′	231 bps
PKM2 R	5′CATGGCAAAGTTCACCCGGA3′	
PKM(C) F	5′TCAG*ATGCTGGAGAGCATGAT*3′*	442 bps
PKM(C) R	5′CTTGCACAGCACAGGGAAGATGCC3′	
	**PHGDH Primers**	
PHGDHSYF	5′AAAGAGGAGCTGATAGCGGA3′	
PHGDHSYR	5′ACCACCTGGAGTTTCTCAGC3′	107 bps
	**SHMT2 Primers**	
SHMT2/SYF	5′CCAGGTCAGGGCTCATCTTC3′	169 bps
SHMT2/SYR	5′TGCTTTAGGGCCACAGCTAC3′	
	**Housekeeping Genes (SYBR Green)**	
HMBSF	5′GGCAACTGTACCTGACTGGA3′	110 bps
HMBSR	5′CTCAGGGCCATCTTCATGCT3′	
B-actinF	5′GAGCACAGAGCCTCGCCTT3′	196 bps
B-actinR	5′CCCACCATCACGCCCTGG3′	
PPIAF	5′GACTGAGTGGTTGGATGGCA3′	77 bps
PPIAR	5′GCTCCATGGCCTCCACAATA3′	

^*^ underlined: 3′exon 7; italic: 5′ exon 8

## Supplementary Materials

Supplementary materials can be found at https://www.mdpi.com/1422-0067/21/5/1661/s1.

## Abbreviations

3-PG	3-phosphoglycerate
ADP	Adenosine Diphosphate
ATCC	American Type Cell Culture Collection
ATF4	Activating Transcription Factor 4
ATP	Adenosine Triphosphate
BrdU	Bromodeoxyuridine
Cal 27	Human Tongue Squamous Carcinoma
Detroit 562	Human Metastatic Pharyngeal Cancer
DMEM	Dulbecco’s Modified Eagle’s Medium
DMSO	Dimethyl Sulfoxide
DNA	Deoxyribonucleic Acid
EDTA	Ethylenediaminetetraacetic Acid
EtdBr	Ethidium Bromide
FaDu	Human Hypopharyngeal Squamous Carcinoma
FBP	Fructose-1,6-bisphosphate
FBS	Fetal Bovine Serum
GAPDH	Glyceraldehyde-3-Phosphate Dehydrogenase
GCN2-ATF4	General Control Nonderepressible 2-activating Transcription Factor 4
gDNA	Genomic DNA
GSH	Glutathione
HIF-1α	Hypoxia-Inducible Factor, Alpha Subunit
HMBS	Hydroxymethylbilane Synthase
HNSCC	Head and Neck Squamous Cell Cancer
HT-29	Colorectal Adenocarcinoma
IL-6	Interleukin 6
mTOR	Mammalian Target of Rapamycin
MTT	Methylthiazolyldiphenyl-tetrazolium bromide
NADH	Nicotinamide Adenine Dinucleotide
NLS	Nuclear Localisation Signal
NRF2	Nuclear Factor (Erythroid-Derived 2)-Like 2
PCR	Polymerase Chain Reaction
PEP	Phosphoenolpyruvate
PHGDH	Phosphoglycerate Dehydrogenase
PI3K	Phosphoinositide 3-kinase
PKM	Pyruvate Kinase M1/2
PPIA	Peptidylprolyl Isomerase A
PPP	Pentose Phosphate Pathway
PSAT1	Phosphoserine Aminotransferase 1
PSPH	Phosphoserine Phosphatase
P-STAT3	Phospho-Signal Transducer and Activator of Transcription 3
RFLP	Restriction Fragment Length Polymorphism
RNA	Ribonucleic Acid
RT-qPCR	Quantitative Reverse Transcription Polymerase Chain Reaction
SHMT2	Serine Hydroxymethyltransferase 2
STAT3	Signal Transducer and Activator of Transcription 3
TBP	TATA-Box Binding Protein
TBS	Tris-Buffered Saline
TBST	Tris Buffered Saline with Tween-20
TMB	3,3′,5,5′- tetramethyl-benzidine diimine
