# Supplementary material for: Cell-Type Specific Metabolic Response of Cancer Cells to Curcumin
Source: Int J Mol Sci. 2020 Feb 28;21(5):1661. doi: 10.3390/ijms21051661 (PMC7084320; doi:10.3390/ijms21051661)
Supplement: Supplementary file 1 [file ijms-21-01661-s001.pdf]

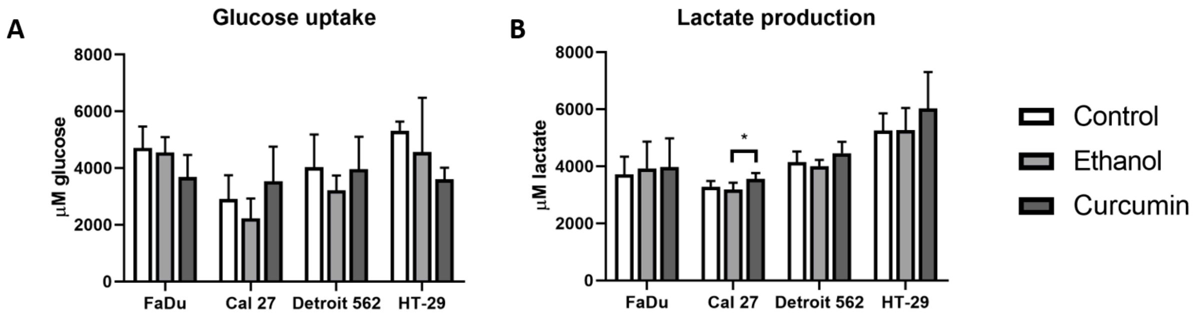

Figure S1. Specific cellular response with respect to the studied metabolic parameters. Glucose uptake (A) and lactate production (B). A one-way ANOVA with Tukey post-hoc test. * $p < 0.05$; ** $p < 0.01$; *** $p < 0.001$; **** $p < 0.0001$.
